# An Updated Review of Recent Advances in Neurosyphilis

**DOI:** 10.3389/fmed.2022.800383

**Published:** 2022-09-20

**Authors:** Jia Zhou, Hanlin Zhang, Keyun Tang, Runzhu Liu, Jun Li

**Affiliations:** ^1^Department of Dermatology, Peking Union Medical College Hospital, Peking Union Medical College, Chinese Academy of Medical Sciences, Beijing, China; ^2^State Key Laboratory of Complex Severe and Rare Diseases, Peking Union Medical College Hospital, Chinese Academy of Medical Sciences, Beijing, China

**Keywords:** neurosyphilis, syphilis, *Treponema pallidum*, cerebrospinal fluid, ocular syphilis, otosyphilis, penicillin, ceftriaxone

## Abstract

Neurosyphilis is caused by *Treponema pallidum* invading the central nervous system, of which the incidence is increasing worldwide. Due to its variable clinical manifestations, diagnosis of neurosyphilis remains challenging, especially the asymptomatic form. This review focuses on recent advances in neurosyphilis, including epidemiology, clinical manifestations, laboratory findings, comorbidities, diagnosis, treatment, prognosis, and basic research. The expansion of men who have sex with men and the infection of human immunodeficiency virus mainly accounted for the increasing incidence of neurosyphilis. The rate of some historically described forms of neurosyphilis in the pre-antibiotic era declined significantly; atypical features are more prevalent. Neurosyphilis, regarded as a great mimicker for neuro-ophthalmic, audio-vestibular, and psychiatric disorders, often presents concomitantly with other diseases, including metabolic disorders. Studies on long non-coding RNAs, miRNAs, chemokines, and metabolites in peripheral blood and cerebrospinal fluid may facilitate exploring the pathogenesis and identifying novel biomarkers of neurosyphilis. The drug resistance of *Treponema pallidum* to penicillin has not been reported; ceftriaxone was proposed to be more effective than penicillin, whereas few randomized controlled trials supported this view. This study may pave the way for further research, especially the diagnosis and treatment of neurosyphilis.

## Introduction

Syphilis, caused by the infection of *Treponema pallidum* (*T. pallidum*), can progress to neurosyphilis at any time after the initial infection. Due to the widespread use of antibiotics, the reported cases of neurosyphilis are less than those in the pre-antibiotic era. However, there has been a resurgence in recent years (1). Supporting evidence is anticipated for the diagnosis and treatment of neurosyphilis, and new research progress has been made recently (2, 3). Therefore, this article reviews recent advances in neurosyphilis, including epidemiology, clinical manifestations, laboratory findings, comorbidities, diagnosis, treatment, prognosis, and basic research.

## Epidemiology

In 2012, 18 million cases of syphilis were reported worldwide, among which 3,50,000 cases caused adverse pregnancy outcomes (4). The incidence continues to rise due to the expansion of susceptible populations, including men who have sex with men (MSM) and people living with HIV (PLWH) (5). The rate of neurosyphilis in PLWH is about twofold as in the immunocompetent population (6). Although the incidence of syphilis is low among women in the USA, it increased by 30.0% in 2018–2019; as for congenital syphilis, the incidence has also been increasing since 2013. In China, from 1990 to 2017, the incidence of syphilis increased from 0.9 to 34.49 per million, second only to viral hepatitis and tuberculosis among infectious diseases. The increasing trend aligned with that of neurosyphilis (7). Male, MSM, advanced age (≥45 years), PLWH without combined antiretroviral therapy, drug use disorder, lack of antisyphilitic therapy, reinfection after a previous syphilis infection, and patients in serofast state are risk factors for neurosyphilis, especially the late forms (8–11). Elevated serum and cerebrospinal fluid (CSF) rapid plasma reagin (RPR) titer, CSF protein concentration, and increased burden of the cerebral small vessel diseases may indicate the aggravation of neurological symptoms (10, 12, 13). Specific subtypes of *T. pallidum* are more likely to induce neurosyphilis; for instance, subtype 14d is the most common neurosyphilis-related genotype in China (14). Specific genes the hosts carry also determine their susceptibility of neurosyphilis, including some single nucleotide polymorphisms on Toll-like receptor (TLR)1, TLR2, TLR6, and the interleukin 10 (IL-10) promoter (15, 16).

Some novel characteristics of neurosyphilis have also been observed nowadays. Currently, the most common form of neurosyphilis is asymptomatic neurosyphilis and meningoencephalitis, although meningovascular syphilis had the highest incidence in some cohort studies (17). The incidence of prevalent symptoms in the pre-antibiotic era, such as tabes dorsalis, dementia paralytica, and gummatous neurosyphilis declined dramatically, as the widespread use of antibiotics in other diseases might indirectly treat neurosyphilis, and timely diagnosis and treatment shortened the course of the disease (18). Recent studies also reported an increase in the incidence rate of ocular syphilis and otosyphilis (19). However, molecular typing showed little evidence of a type specifically causing ocular syphilis. Thus, this increase might just reflect a resurgence in total syphilis cases (20).

## Clinical Manifestations and Laboratory Findings

Based on the disease progression, neurosyphilis can be divided into early and late stages. Manifestations that do not fall into either of the two stages are classified as atypical neurosyphilis. Early neurosyphilis can be classified as asymptomatic and symptomatic forms, and the symptomatic form includes symptomatic meningitis and meningo-vasculitis. The manifestations of late neurosyphilis include dementia paralytica and tabes dorsalis. Patients with early neurosyphilis are more likely to have ocular involvement than those of late stage (18). Cerebral small vessel disease, one of the major causes of cognitive impairment, is more prevalent in patients of late stage (12). These forms were detailed in the following sections.

Asymptomatic neurosyphilis is defined as a neurologically asymptomatic form with serological and CSF abnormalities, typically occurring in the early months of infection. Patients can be diagnosed with the following features, CSF lymphocytosis (<100 cells/μL), elevated protein concentration (<100 mg/dL), a reactive result of cerebrospinal fluid-Venereal Disease Research Laboratory (CSF-VDRL) assay, or a combination of these abnormalities. However, these existing benchmarks cannot be directly used in PLWH, for HIV itself may trigger CSF lymphocytosis and elevated protein concentration.

As a common manifestation of early neurosyphilis, symptomatic meningitis often appears within the first year of infection, in the form of headache, nausea, vomiting, and neck pain, accompanied by ocular involvement like inflammation and blurry vision. It can further lead to cranial neuropathy, meningo-vasculitis, and damage to the brain parenchyma. CSF abnormalities in early symptomatic meningitis are more severe than those in asymptomatic forms, with lymphocyte count around 200–400 cells/μL, protein concentration around 100–200 mg/dL, and CSF-VDRL tests almost always reactive (6). Neuroimaging profile usually demonstrates enhancement of CSF, meninges, or cranial nerves in this stage. Meningo-vasculitis can cause thrombosis, ischemia, and infarction. For young patients with strokes, syphilitic meningo-vasculitis should be considered, since there was one study showing that 14.09% of patients with neurosyphilis had an ischemic stroke as a primary symptom (21). In southern Brazil, the reactive result of serological tests for syphilis is common in patients with acute stroke (22). As in cases of meningo-vasculitis, CSF white blood cell (WBC) count is usually around 5–74 cells/μL, with protein concentration around 22–101 mg/dL, while CSF-VDRL tests are not always reactive (23). In patients with meningo-vasculitis, focal narrowing, dilatation, and occlusion may be seen in angiography, while neuroimaging may show evidence of infarction. It was reported that neuroimaging progression could continue in symptomatic patients even after standardized antisyphilitic therapy, which was confirmed by a study showing infarction lesions in 42.1%, mild to severe brain atrophy in 47.4%, and white matter demyelination in 15.8% of treated patients in the follow-up (24).

Dementia paralytica and tabes dorsalis are two major forms of late neurosyphilis, usually appearing 15–20 years after the initial infection. The CSF of patients with late neurosyphilis shows only mild lymphocytosis or slightly elevated protein concentration, compared to those with early neurosyphilis. Dementia paralytica presents with neurasthenic syndromes and personality disorders in the early stage, which is difficult to distinguish from Alzheimer's disease (25); however, in the middle and late stages, patients will show physical symptoms, such as Argyll Robertson pupils, optic atrophy, sensory ataxia, tremors, and dysarthria. In those paretic patients, CSF protein concentration ranged from 20 to 186 mg/dL, and CSF WBC count ranged from 0 to 98 cells/μL, with 94.9% of CSF-VDRL tests turned to be reactive (26). Signs of tabes dorsalis include areflexia, urinary retention, sexual dysfunction; 60% of patients in that stage present with Argyll Robertson pupils. In tabetic patients in a cohort study, CSF protein concentration ranged from 14 to 64 mg/dL, and CSF WBC count ranged from 2 to 145 cells/μL (26).

Atypical neurosyphilis is more frequently observed nowadays. The symptoms of early neurosyphilis can mimic most neuro-ophthalmic diseases, while the neurological involvement in the late stage can mimic psychiatric or autoimmune disorders. Rapidly progressive psychosis, such as dementia (27), cognitive impairment (28), and subacute confusion (29), was reported in some cases of late neurosyphilis, some of which were initial clinical presentations. Thus, it is essential to ask for consultation from psychiatrists. Additionally, herpesviral encephalitis, polyradiculoneuropathy, mania, extrapyramidal syndrome, amyotrophic lateral sclerosis, and cauda equina syndrome were also reported in recent cases (28). For patients without other common risk factors but with neuropsychiatric symptoms, screening for syphilis should be recommended.

## Comorbidities

Patients with neurosyphilis may have other diseases, such as HIV infection, posing great diagnostic and therapeutic challenges to clinicians. Recent studies focused on the current status and underlying mechanisms of the comorbidities of neurosyphilis and HIV infection. A study from Missouri reported that 7.4% of HIV-positive MSM patients were diagnosed with primary or secondary syphilis in 2016, compared with 3.1% of immunocompetent MSM patients (30). Syphilis can exacerbate HIV infection and inflammation in the central nervous system, increasing HIV viral load and decreasing CD4 + T lymphocyte count. For HIV-uninfected individuals, syphilitic skin lesions may also provide access to HIV acquisition (31). Meanwhile, HIV-associated immunosuppression can increase susceptibility to neurosyphilis. The diagnosis of neurosyphilis is also more difficult in PLWH. On the one hand, PLWH may have false positive reactions to serological tests for syphilis (32). On the other hand, HIV itself can lead to a slight increase in CSF WBC count and protein concentration (9). Besides, cases of gonorrhea, viral hepatitis, and chlamydia comorbid with syphilis were reported (33). Weathers et al. also showed that comorbid hepatitis and herpes simplex would increase the risk of syphilis (34).

Ocular syphilis and otosyphilis were discussed as comorbidities of neurosyphilis here. Ocular syphilis, occurring at any stage of syphilis, is becoming an important cause of uveitis. Furtado et al. found that the most common form of intraocular inflammation induced by ocular syphilis was posterior uveitis, followed by panuveitis (35). Diminished vision, interstitial keratitis, optic neuropathy, and retinal vasculitis are typical manifestations, and ocular hypertension and cataract were also reported (36). Mathew et al. found that 37% of patients with ocular syphilis had neurosyphilis (37). The frequency of neurosyphilis comorbid with ocular syphilis was significantly higher in PLWH than in immunocompetent patients (37). In one study on PLWH with comorbid neurosyphilis, those with ocular syphilis were more likely to have elevated CSF WBC count than those without ocular syphilis (38). Due to these possible misleading symptoms, ocular syphilis was sometimes misdiagnosed as, for example, retinal detachment or vitreous hemorrhage. Delayed management of ocular syphilis can cause poor vision in the follow-up (39). Otosyphilis is also a mimicker for many audio-vestibular disorders, occurring at any stage of syphilis. The main manifestation is sensorineural hearing loss, which will develop into conductive hearing loss. Other common manifestations include vertigo, tinnitus, and gait instability (40); accompanying meningitis and ocular involvement were also reported (41). Some studies suggest that *T. pallidum* may impair the eighth cranial nerve, temporal bone, and cochleovestibular apparatus (42). Regardless of the CSF profile, otosyphilis and ocular syphilis should be treated with the same protocols for neurosyphilis (43).

Patients with neurosyphilis might have specific metabolic characteristics. The incidence of hyperlipidemia and hypertension in patients with neurosyphilis was 21.4% and 45.9%, respectively, both significantly higher than that in patients with other neurological infections. Patients with neurosyphilis also presented with significantly higher levels of low-density lipoprotein, total cholesterol, systolic and diastolic blood pressure (44). Glycated hemoglobin A1c (HbA1c) is an indicator of diabetes and neurosyphilis, playing an unclear role in blood-brain barrier (BBB) disruption, an essential step in the progression of both diseases (45).

## Diagnosis

The diagnosis of neurosyphilis is based on serum and CSF tests, which can be further classified as treponemal and nontreponemal tests. Nontreponemal serum tests mainly include rapid plasma reagin (RPR) and venereal disease research laboratory (VDRL); treponemal serum tests mainly include *T. pallidum* particle agglutination assay (TPPA), *T. pallidum* enzyme immunoassay (TP-EIA), chemiluminescence immunoassay, and fluorescent treponemal antibody absorption (FTA-ABS). For patients with ocular or neurological symptoms, features of tertiary syphilis, or serofast status, lumbar puncture and CSF tests are needed for the diagnosis of neurosyphilis. CSF tests mainly include CSF WBC count, CSF protein concentration, CSF-RPR, CSF-TPPA, and CSF-FTA-ABS. As for nontreponemal tests, the sensitivity varies by the progress of neurosyphilis; for patients with late neurosyphilis, nontreponemal tests are of a high false-negative rate (46).

Conventional diagnostic methods of neurosyphilis face some challenges. The diagnosis relies heavily on CSF-VDRL, of which the specificity is high (89–96%), but the sensitivity is relatively low (12–48%) (47). Alternative methods, such as CSF toluidine red unheated serum test (CSF-TRUST), might not perfectly remedy the low sensitivity; CSF chemiluminescence immunoassay was preferred, with the sensitivity of 99.57–100%, and the specificity of 99.81–99.88% (38). Some studies suggested chemokine (C-X-C motif) ligand (CXCL) 13 as a promising indicator, with specificity from 76 to 81% and sensitivity from 78 to 83% (47–49). However, the optimal benchmark for CXCL13 remains controversial. CSF samples from cohorts are needed to establish an appropriate cut-off for the diagnosis of neurosyphilis (47). The combination of CXCL8, CXCL10, and CXCL13 might be a better indicator with specificity of 92.9% and sensitivity of 90.4% (50). CSF WBC count, protein concentration, and clinical manifestations should all be considered along with CSF-TPPA; the sensitivity of this combined diagnostic method was up to 98% (51). Adjusting the cut-off of tests was also proposed as a solution to low specificity and sensitivity. Serum TPPA, commonly used in patients with unknown prior history of syphilis, was recommended to enhance the specificity of CSF test, when the titer cut-off was adjusted to >1:320. For those with high suspicion of neurosyphilis but without a positive result of CSF VDRL test, the CSF treponemal assay like CSF TPPA was a reasonable recommendation to reduce the false-negative rate (52). Polymerase chain reaction (PCR) and immunoblot were recommended as new automated treponemal tests. A study showed that the specificity and sensitivity of PCR ranged from 60 to 100% and 40 to 70%, respectively; as for immunoblot, the specificity varied from 91.7 to 92.0%, and the sensitivity varied from 93.8 to 100% (48). Nowadays, automated treponemal tests are often accompanied by a nontreponemal test as a following confirmatory step, which is called the reverse-sequence algorithm and has shown ideal effects in the low-prevalence populations (53). Consequently, the diagnosis should make full use of serum and CSF samples by a combination of several tests in order to obtain a convincing result.

Conventional benchmarks may not be convincing for neurosyphilis patients coinfected with HIV, because HIV may cause CSF lymphocytosis and elevated protein concentration. Due to the difficulty in diagnosing asymptomatic neurosyphilis in PLWH, lumbar puncture was recommended by some experts for all syphilis patients coinfected HIV, which might also cause unnecessary costs and risks. CDC suggested that lumbar puncture should be given to PLWH with neurological signs or symptoms, and/or serofast status, and/or tertiary syphilis, which was supported by another cohort analysis (30).

Recently, the point-of-care syphilis tests were gradually popularized, due to the epidemic of syphilis in low-and middle- income countries. Low-and middle-income countries also showed high prevalence rates, long diagnostic delays, low follow-up rates, and inaccessibility to trained clinicians and necessary facilities (54). Optimized commercial immunochromatographic strip tests, such as the CSF-Syphicheck and the DPP Chembio syphilis assay, showed satisfying diagnostic specificity and sensitivity compared to CSF-VDRL (62–64% sensitivity, 79–81% specificity in CSF-Syphicheck; 63–92% sensitivity, 85–100% specificity in DPP Chembio syphilis assay) (55, 56). Though further evaluation is needed, point-of-care tests may be widely applied in resource-limited regions and contribute to the control of sexually transmitted diseases, especially for neurosyphilis and congenital syphilis.

## Treatment and Prognosis

Treatment guidelines for neurosyphilis released by the United States, the United Kingdom, Europe, and China are outlined in Table 1. They all highlighted that PLWH should be treated as immunocompetent patients, and ceftriaxone might be an alternative treatment to penicillin. A divergence of views lay on the adoption of steroids in patients with neurosyphilis, which was only recommended in some of the guidelines. Efficacy of different therapies is always a major concern, whereas it is hard to draw a definite conclusion due to those low-quality, small-size, and highly-biased trials (58, 59). A cohort study including 208 patients with neurosyphilis during 1997–2017 showed serological cure rates of 88% in the ceftriaxone group and 82% in the penicillin G group (2), while another study showed clinical cure rates of only 44% in the ceftriaxone group and 18% in the penicillin G group (59). Meanwhile, neurological sequelae were reported in 41.8% of the neurosyphilis patients in a retrospective study (60). PLWH coinfected with neurosyphilis were more likely to fail the intravenous penicillin G therapy (61, 62).

**Table 1:** Treatment recommendations for neurosyphilis.

**Guidelines**	**Treatment modalities**
Guidelines from Centers for Disease Control and Prevention of the United States (6)	IV^#^ aqueous crystalline penicillin G 3–4 million U every 4 h, or continuous infusion 18–24 million U/d for 10–14 days.
The United Kingdom National Guidelines (52)	IM^*^ procaine penicillin G 1.8–2.4 million U/d, following with oral probenecid 500 mg every 6 h for 2 weeks; or IV penicillin G 1.8–2.4 g every 4 h for 2 weeks.
European Guidelines (43)	IV penicillin G 18–24 million U/d, as 3–4 million U every 4 h for 10–14 days; or IV ceftriaxone 1–2 g/d for 10–14 days; or IM procaine penicillin G 1.2–2.4 million U/d, following with oral probenecid 500 mg every 6 h for 10–14 days, when IV penicillin G cannot be used.
Guidelines from Chinese Center for Disease Control and Prevention (57)	IV aqueous crystalline penicillin G 18–24 million U/d for 10–14 days, if necessary, following with IM benzathine penicillin G 2.4 million U/week for 3 doses; or IM procaine penicillin 2.4 million U/d and oral probenecid 500 mg every 6 h for 10–14 days, if necessarily, following with IM benzathine penicillin G 2.4 million U/week for 3 doses. Second-line therapy option: IV ceftriaxone 2 g/d for 10–14 days. For penicillin-allergic patients, oral doxycycline 100 mg twice a day, for 1 month.

# *IV = intravenous.*

* *IM = intramuscular*.

Penicillin is preferred worldwide as front-line therapy for neurosyphilis, and the drug resistance of *T. pallidum* to penicillin has not been reported (63). Some studies found that ceftriaxone was more effective than penicillin, but few large randomized controlled trials supported this view (2, 59, 60). Tetracycline, erythromycin, and chloramphenicol have all been applied to treat syphilis, whereas erythromycin and chloramphenicol were reported to induce intestinal dysbiosis and irreversible aplastic anemia, respectively (59). The treatment effects of penicillin and doxycycline were similar in some series (64); doxycycline is cheaper yet forbidden in pregnant women. The only treatment modality certified for pregnant women with syphilis is parenteral penicillin G. The concurrent administration of probenecid is widely used in antibiotic therapy for neurosyphilis. A recent study proved that oral amoxicillin with probenecid could elevate the drug level in CSF (43). However, oral probenecid with intramuscular (IM) procaine penicillin should be carefully used in HIV-positive MSM (65).

The Jarisch-Herxheimer reaction (JHR) may occur within the first 24 h of neurosyphilis treatment, presenting as headache, chills, fever, rashes, and myalgias, of which the mechanism is unclear (66). Commonly used antibiotics, including penicillin, erythromycin, azithromycin, tetracyclines, clarithromycin, and levofloxacin, could all cause JHR (67). JHR usually occurs among patients with early syphilis, as the risk of JHR increases by 19% for every twofold increase in RPR titer, suggesting that higher spirochete load may cause a significantly higher risk of JHR (68). For PLWH, particularly those with encephalitis, JHR may be more severe (28). Steroids, like prednisolone, are routinely used to prevent JHR; TNF-α antibodies and acetaminophen were also used in some cases (69).

Serofast, a lack of serological response to antisyphilitic therapy, is currently a major concern for clinicians. This clinical scenario occurred in over one-third of patients, especially those with lower test titers or late latent syphilis (70). A successful response to antisyphilitic therapy is defined as a fourfold dilution decline in CSF-VDRL and the normalization of CSF WBC count, except for PLWH. Patients with serofast only show a decrease less than fourfold in non-treponemal antibody titers after a specific period of time (6 months for early-stage syphilis; 12 months for late-stage syphilis), or show low titers consistently after treatment (6). The cause of serofast is still unclear. Possible explanations of the lack of serological response to antisyphilitic therapy include that the patients have undiagnosed neurosyphilis and fail to respond to routine therapy; patients have comorbid HIV, malignancies, or immune diseases; and patients are re-infected by having sex with others (71, 72). Prediction of the serofast occurrence is still difficult, and retreatment to serofast patients can not significantly improve the cure rates (73, 74).

Several factors influence the prognosis of patients with neurosyphilis, which can also be used as indicators to assess the therapeutic effect. Although HIV coinfection may confound the diagnosis of neurosyphilis, the prognosis of patients does not differ by the HIV infection status (75). Diplopia and severe atrophy of brain parenchyma were proved to be associated with poor prognosis. Headache often contributes to an early diagnosis of disorders in the central nervous system, which is a favorable prognostic factor in patients with neurosyphilis (60); the changes in electroencephalogram and metabolites in CSF may be auxiliary indicators of the prognosis. Jiang et al. found that the electroencephalogram Lempel-Ziv complexity (EEG-LZC) value after treatment was significantly higher than that before treatment, suggesting that neuron reconnection would improve the brain function (76). In addition, CXCL13, tau, neurogranin, and metabolites in CSF could also be used to evaluate the cognitive function of patients with neurosyphilis and the effectiveness of antisyphilitic therapy (25, 77).

Reinfection of syphilis, which can start with the latent or tertiary stage (78), is more prevalent in MSM and PLWH (79, 80). A retrospective study on MSM in California from 2002 to 2006 showed that 5.9% of MSM had repeat infection of syphilis within 2 years after the first infection (80). Another 8-year surveillance in Brazil showed that 13.6% of patients had reinfection with *T. pallidum* (81). A four-fold drop in serum RPR titer of patients within 6–12 months is defined as the serological cure. However, some patients have a rise in RPR titer within the first 12 months, for which, the main reason is usually reinfection, rather than recurrence (82). Molecular studies are necessary when the corroborative history of patients is inaccessible to determine whether the rise in RPR titer is due to reinfection or recurrence (83). The mechanisms of reinfection are related to the destruction of adaptive immunity by *T. pallidum* (84). An immunodominant-evasion model further suggested that the TprK, an outer membrane protein with prolific antigenic variation, explained the ability of *T. pallidum* to escape the adaptive immunity (84).

## Basic Research

A major obstacle for the research on *T. pallidum* is that it can rarely be cultured continuously *in vitro*. Recent basic research on neurosyphilis mainly focuses on the pathogenic mechanisms of neurosyphilis, and the potential biomarkers for diagnosis or prognosis monitoring.

Research in the pathogenic mechanisms has proved that *T. pallidum* crosses the endothelial barriers through interjunctional penetration, disseminating throughout the body and invading various organs (85). This process depends largely on the outer membrane proteins of *T. pallidum*, such as Tp92, to promote the adhesion and proliferation of spirochetes, enhance the permeability of endothelial cells, and immunomodulate in the inflammatory responses (86). *T. pallidum* can activate both humoral and cellular immunity by antigens such as *T. pallidum* repeat protein (Tpr), whereas cellular immunity plays a major role in eliminating pathogens but is also significantly suppressed in the late stage of infection (87). The immune escape mechanism of *T. pallidum* has been well studied. Due to the lack of exposed lipoproteins on the outer membrane of *T. pallidum*, antigen-presenting cells are more difficult to contact the pathogen-associated molecular patterns and unable to activate the innate immune system (88). The common targets of humoral immune response are the variable region of membrane proteins such as TprK (89). However, it was reported that the continuous mutation of the TprK gene, especially the variable region, enabled *T. pallidum* to escape the immune response and caused persistent infection (90). Qin et al. reported that in syphilis patients in serofast state, unbalanced T cell subsets could suppress the cellular immunity with decreased levels of CD4+ T cells, decreased ratio of T helper (Th)1/Th2 cells, and increased levels of CD8+ T cells (91). Another study found increased levels of regulatory T cells and transforming growth factor-β in peripheral blood of the serofast patients with secondary syphilis, which was mainly induced by the antigen TpF1 and might suppress the cellular immunity (92).

Host immune response also plays an important role in the pathogenesis of neurosyphilis, mainly in the form of T cell-mediated delayed-type hypersensitivity (87). Recent studies showed a significantly elevated levels of IL-8, CC chemokine ligand 20 (CCL-20) in serum, elevated levels of CXCL13, interferon(IFN)-γ in CSF, and elevated levels of IL-6, IL-10, IL-17 in both serum and CSF of neurosyphilis patients (49, 93–96). While the imbalance between Th17 and Th1 had been proved to aggravate inflammation in patients with secondary syphilis, how neurosyphilis developed in syphilis patients and the role host immune response played in the development of neurosyphilis remained unclear (97). The latest research proposed that CXCL13/CXCR5 mediated the accumulation of B cells and immunoglobulin G in the CSF of neurosyphilis patients (98); IL-17 not only mediated the inflammatory response, but also activated endothelial contraction and destroyed the tight junctions of the BBB (99); IL-8 and CCL-20 induced by TpF1 could induce vascular inflammation and angiogenesis (100). Liu et al. found that, compared to syphilis/non-neurosyphilis patients, patients with symptomatic neurosyphilis showed a significantly increased expression of CD8+ IFN-γ+ cell but decreased expression of CD8+ IL-17+ cell in the peripheral blood, which might also explain the pathogenesis of symptomatic neurosyphilis (96).

Recent studies also focused on the mechanisms of neurological damage caused by *T. pallidum. T. pallidum* can invade the central nervous system since the early stage of syphilis; however, the relationship between *T. pallidum* and the BBB remains unclear. Neurosyphilis, especially in the stage of general paresis, is similar to Alzheimer's disease. Thus, Alzheimer's-associated biomarkers are widely studied in neurosyphilis patients with cognitive impairment. Receptors expressed on myeloid cells 2 (sTREM2), neurofilament light proteins, and phosphorylated neurofilament heavy subunit were elevated in CSF of patients with neurosyphilis, suggesting that activated microglia were associated with neuron damage caused by *T. pallidum* (101, 102). β-amyloid precursor protein cleaving enzyme (BACE1) and neurogranin also increased in the CSF of patients with neurosyphilis, indicating that the synaptic destruction would appear in the stage of general paresis (25). CXCL13 was also involved in CSF pleocytosis and neurological damage (49, 103).

Studies on RNA, proteins, and metabolites in peripheral blood and CSF have facilitated the discovery of novel indicators for neurosyphilis. Long non-coding RNAs expressed in peripheral blood of patients with neurosyphilis were emphasized in recent studies. IFN-γ production was proved to be mediated by the interaction between lncRNA-ENST00000421645 and PCM1 (104). miRNAs in exosomes released by neuroglial cells could be potential biomarkers in CSF of patients with neurosyphilis for brain parenchymal damage, including miR-570-3p, miR-590-5p, miR-570-5p, and miR-21-5p (105). The elevated level of chemokines was associated with inflammatory disorders in the central nervous system (50); CXCL13, CXCL8, and CXCL10 were suggested as novel biomarkers with high sensitivity (49). Interleukin-10 in CSF was also regarded as a biomarker of neurosyphilis, especially of asymptomatic neurosyphilis (93). In addition, metabolomics studies recently identified representative metabolites in CSF of patients with neurosyphilis as new indicators, such as L-histidine, bilirubin, alpha-kamlolenic acid, prostaglandin E2, palmitoyl-L-carnitine, butyryl-L-carnitine, D-mannose, L-gulono-gamma-lactone, hypoxanthine, and N-acetyl-L-tyrosine (77, 106).

## Discussion

As the folk goes, “One night with Venus, a lifetime with Mercury”. The everlasting stigmatization of syphilis has hindered the development of diagnostic and therapeutic management, especially in the follow-up, partner notification, and wide-range screening in susceptible populations (107). As a so-called prevalent disease in celebrities with a libertine lifestyle, syphilis was said to have infected the most influential people like Shakespeare and Columbus, and evoked further panic in Europe by the horrible symptoms of mercury poisoning during treatment (108). Mystified and misread for centuries, syphilis was ultimately controlled by the wide use of antibiotics. However, it has not been eliminated till now, due to the increase in sexual risk behaviors mainly in homosexual communities since the mid-1990s (109). Neurosyphilis, a stage that *T. pallidum* invades the central nervous system, may progress to severe cardiovascular complications and irreversible neurological damages if treatment is delayed. The alarming resurgence of syphilis also attracts attention to the incidence of neurosyphilis, particularly in MSM and PLWH.

The latest research progress on neurosyphilis in recent years is summarized. In some series, neurosyphilis was diagnosed disproportionately in young patients with ischemic stroke, which argued for the screening in low-risk populations presenting with specific abnormalities, including ischemic stroke, rapidly progressed neuropsychiatric symptoms, and recurrent ocular inflammation (110). However, the implementation of screening for sexually transmitted infections may bring humiliation to the subjects, damage their close relationships and personal image in society (53). Comorbid neurosyphilis with HIV or other sexually transmitted infections has been reported worldwide, of which the comorbidities may cause misdiagnosis and failure of conventional therapy; HIV coinfection does not affect the prognosis of patients with neurosyphilis (75). Most of the basic research focused on the pathogenesis of neurosyphilis, based on the changes of non-coding RNAs, chemokines, proteins, lymphocyte subtypes, and metabolites in CSF of patients with neurosyphilis. These potential biomarkers may be promising for diagnosis and disease monitoring, but whether they can explain the pathogenesis of neurosyphilis remains unclear. Evidence showed similarity between neurodegenerative diseases and neurosyphilis in pathogenesis and clinical manifestations, which suggested that the existing theories of Alzheimer's disease might enlighten the research of neurosyphilis (25). The epidemic of syphilis and neurosyphilis in low- and middle- income countries is difficult to control due to the resource-limited settings there. However, point-of-care syphilis tests developed in recent years may relieve the pressure of disease control for its feasibility and inexpensiveness (56). JHR has also been reported in almost all types of commonly used antibiotics for syphilis, especially in the treatment of PLWH (68).

There are still some problems to be solved. (1) Due to the inaccessible *in-vitro* culture and unknown host response to the infection of *T. pallidum*, the development of vaccines still has a long way to go (111). (2) The false-negative and false-positive rates remain high in a single treponemal or nontreponemal test, thus combined use of various diagnostic methods is recommended, alongside neuroimaging and individuals' history of sex life. It should be noticed that not only false-negative tests may delay the treatment, but false-positive tests may also cause unnecessary management and stigmatization to patients. (3) Penicillin remains the first choice for the treatment of neurosyphilis, but there are few alternative treatment modalities for patients with cross-allergy of penicillin and ceftriaxone (59). Some conventional therapies also have side effects for PLWH (65). (4) Another challenge in the treatment is serofast. The cause of the serofast is still unclear and retreatment to serofast patients can not significantly improve the cure rates (71, 72). Prediction of the serofast occurrence is also difficult. Moreover, some serofast patients may no longer be threatened by syphilis, but other patients may still be at risk, which is hard to evaluate. (5) Lumbar puncture also remains controversial in the diagnosis of neurosyphilis, due to its difficulty of implementation and potential risks posed to patients. CDC does not recommend lumbar puncture for PLWH with syphilis yet without neurological symptoms, and only one lumbar puncture is allowed every 6 months after treatment to monitor the prognosis. Thus, patients with neurosyphilis are easily lost to follow-up. (6) Additionally, the ignorance of patients about the symptoms of early syphilis or asymptomatic neurosyphilis is common and often results in delayed diagnosis and treatment, which might account for the rising incidence of neurosyphilis. A study from Tanzania found that the average consultation delay for rural patients was almost double that for urban patients (112). Another study demonstrated that only 16.7% patients with symptomatic neurosyphilis visited dermatology department, as patients rarely paid attention to their skin conditions (24). (7) Some essential approaches for controlling neurosyphilis are still time-consuming and difficult to undertake, including public education, partner notification, and screening projects. Whereas, excessive screening and prophylactic treatment of syphilis may bring humiliation to the individuals and waste of resources to the society. Thus, when developing new screening or prevention policies, balance should be maintained carefully. For instance, prophylactic treatment is not recommended for partners of neurosyphilis patients due to the limited infectiousness of neurosyphilis, whereas serological tests are necessary for them to exclude the risk of transmission (54). Further research could concentrate on the pathogenesis, standards and guidelines, treatment modalities, JHR and serofast, follow-up, and the prevention of large-scale coinfection in MSM and PLWH.

## Conclusions

This article reviews recent advances in neurosyphilis, including epidemiology, clinical manifestations, laboratory findings, comorbidities, diagnosis, treatment, prognosis, and basic research. The incidence of neurosyphilis has been increasing in recent years, mainly in MSM. The incidence of historically described forms of neurosyphilis in the pre-antibiotic era declined significantly nowadays, with atypical features becoming more common. Neurosyphilis can mimic most neuro-ophthalmic, audio-vestibular, and psychiatric disorders. Comorbidities of neurosyphilis are also prevalent and variable, and patients may present with specific metabolic characteristics. Clinical diagnostic methods remain immature, whereas recent studies on long non-coding RNA, miRNA, chemokines, and metabolites in peripheral blood and CSF may facilitate the research on the pathogenesis and new indicators of neurosyphilis. The resistance of *T. pallidum* to penicillin has not been discovered. Some studies also found that ceftriaxone was more effective than penicillin, but few large randomized controlled trials supported this view. Attention should also be paid to specific populations, such as PLWH, pregnant women, and those allergic to penicillin.

## Author Contributions

JZ, HZ, and KT reviewed the literature and wrote the manuscript. RL reviewed the literature. JL revised the manuscript. All authors meet the International Committee of Medical Journal Editors (ICMJE) criteria for authorship for this article, take responsibility for the integrity of the work as a whole, and have given their approval for this version to be published.

## Funding

The Rapid Service Fee was funded by Peking Union Medical College Hospital. We acknowledge the support from Beijing Municipal Science and Technology Commission (No. Z191100006619011), the Capital's Funds for Health Improvement and Research (2020-2-4016), and CAMS Innovation Fund for Medical Sciences (CIFMS 2020-I2M-C&T-B-048).

## Conflict of Interest

The authors declare that the research was conducted in the absence of any commercial or financial relationships that could be construed as a potential conflict of interest.

## Publisher's Note

All claims expressed in this article are solely those of the authors and do not necessarily represent those of their affiliated organizations, or those of the publisher, the editors and the reviewers. Any product that may be evaluated in this article, or claim that may be made by its manufacturer, is not guaranteed or endorsed by the publisher.
